# Clustering-Driven DGS-Based Micro-Doppler Feature Extraction for Automatic Dynamic Hand Gesture Recognition

**DOI:** 10.3390/s22218535

**Published:** 2022-11-05

**Authors:** Chengjin Zhang, Zehao Wang, Qiang An, Shiyong Li, Ahmad Hoorfar, Chenxiao Kou

**Affiliations:** 1 Beijing Key Laboratory of Millimeter Wave and Terahertz Technology, Beijing Institute of Technology, Beijing 100081, China; 2 Key Laboratory of Microwave Remote Sensing, National Space Science Center, Chinese Academy of Sciences, Beijing 100190, China; 3 Department of Biomedical Engineering, Fourth Military Medical University, Xi’an 710032, China; 4 Antenna Research Laboratory, Center for Advanced Communications, Villanova University, Villanova, PA 19085, USA

**Keywords:** dynamic hand gesture recognition, sparse signal representation, dynamic group sparsity, micro-Doppler features

## Abstract

We propose in this work a dynamic group sparsity (DGS) based time-frequency feature extraction method for dynamic hand gesture recognition (HGR) using millimeter-wave radar sensors. Micro-Doppler signatures of hand gestures show both sparse and structured characteristics in time-frequency domain, but previous study only focus on sparsity. We firstly introduce the structured prior when modeling the micro-Doppler signatures in this work to further enhance the features of hand gestures. The time-frequency distributions of dynamic hand gestures are first modeled using a dynamic group sparse model. A DGS-Subspace Pursuit (DGS-SP) algorithm is then utilized to extract the corresponding features. Finally, the support vector machine (SVM) classifier is employed to realize the dynamic HGR based on the extracted group sparse micro-Doppler features. The experiment shows that the proposed method achieved 3.3% recognition accuracy improvement over the sparsity-based method and has a better recognition accuracy than CNN based method in small dataset.

## 1. Introduction

Over the last decade, dynamic hand gesture recognition (HGR) has received an increasing research interests for human-machine interactions (HMI). It possesses great significance in a number of short-range contactless applications [1,2,3,4,5,6]. Getting rid of the physically contact sensors required in traditional electromyography (EMG)-based or glove-based HGR tasks [7,8,9] brings many benefits, including accessibility to all potential users (healthy persons, patients with limited mobility, those allergic to contact sensors), convenience of long-term monitoring (enabling automatic stop and go detection), mobility and flexibility of deployment (adaptation to all environmental and lighting conditions).

Various schemes have been proposed for non-contact dynamic hand gesture recognition, such as optical sensors [10,11,12], acoustic sensors [13], Wi-Fi [14,15], and radar-based methods [16]. Radar-based HGR has attracted a considerable attention and tremendous progress has been made since it can work in all lighting conditions, even in penetrating condition, and with a privacy preserving manner. The micro-Doppler features extracted from the spectrograms obtained using the short-time Fourier transform (STFT) analysis, are often utilized to characterize different hand gestures. In addition, some studies used wideband radar or multi-antenna radar systems to obtain distance or angle information [17,18,19]. Introducing more perspectives improves the ability of recognizing gestures in certain scenarios, such as using angle information to distinguish between flapping the hand to left and right or rotating the hand clockwise and counterclockwise.

Radar-based HGR task differs from the arm motion recognition [20,21], which has also grown rapidly in recent years. Arm motions are performed with the joint participation of the upper arm, the lower arm and the palm. With the involvement of the upper arm, a quite pronounced time-frequency distribution can be achieved due to the wide motion spreading range, rapid velocity changes, and large radar cross section (RCS) of the upper arm. Unlike arm motions, hand gestures involve the motions of the fingers, the palm and the lower arm. The motion expansion, the speed and the RCS of the hand gestures are much smaller than those of the arm motions. As a result, the motion features of hand gestures are strongly attenuated and degenerated, which make the recognition much more difficult than that of the arm motions. Thus, there is an urgent need to investigate more effective ways of conducting feature extraction and enhancement for hand gesture recognition.

The lower dimensional features are attractive for recognition due to only small amount of data needed for classifier in comparison with the methods based on neural network. In refs. [22,23,24,25], various handcrafted features are extracted from time-frequency maps and used for HGR. Eigenspace features are also commonly used for HGR [26]. In ref. [3], application-specific features extracted using principal component analysis (PCA) were utilized to recognize dynamic hand gestures. The sparse reconstruction-based feature extraction approach has also achieved good performance and proven to be effective to handle with the gesture recognition task [27,28,29]. However, the approach only considered the fully sparse property. In fact, the micro-Doppler signatures of hand gestures exhibit a more important feature, that is the local clustering.

In this paper, in the aim of further enhancing hand motion features and improving the recognition performance, we proposed a novel strategy to jointly consider the sparsity and clustering properties of the micro-Doppler signatures and used a dynamic group sparsity (DGS) model [30] to extract corresponding features. Firstly, the relationship between the radar echoes of hand gestures and their corresponding micro-Doppler signatures are established using a time-frequency dictionary. Secondly, the micro-Doppler features are modeled using structured priors and extracted using the DGS-Subspace Pursuit (DGS-SP) algorithm [30]. Then, the features are fed into an SVM classifier. At last, experiments with data collected by a 24 GHz continuous wave (CW) radar are carried out to verify the efficacy of the proposed method. The results demonstrate that the structured feature is beneficial to improve the accuracy of dynamic hand gesture recognition.

The remainder of this paper is organized as follows. In Section 2, the DGS-based structured sparse model and the DGS-SP-based feature extraction algorithm are detailed. In Section 3, the dynamic hand gesture experiments are implemented, and the recognition accuracy is presented to verify the effectiveness of the proposed method in comparison with the sparse only method and the convolutional neural network (CNN) method. Section 4 summarizes the paper.

## 2. Materials and Methods

### 2.1. Micro-Doppler Signatures of Dynamic Hand Gesture

**Figure 1:**
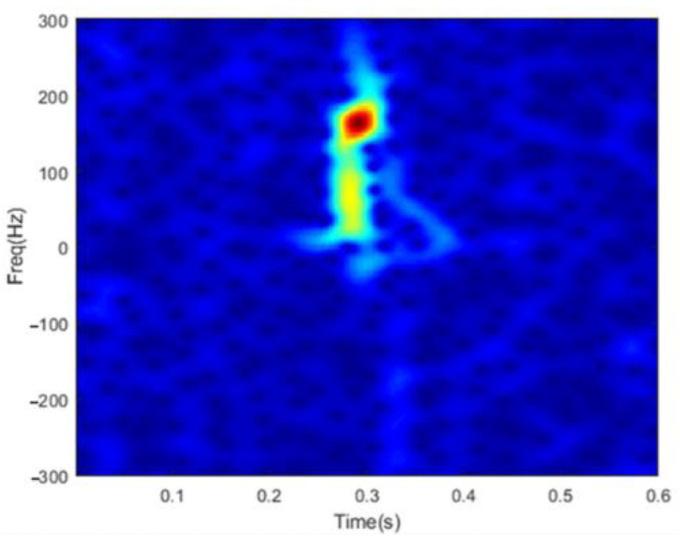
An example spectrogram.

Time-frequency analysis is the most common approach to conduct motion recognition task. Usually, the short-time Fourier transform (STFT) is applied to process the radar records so as to obtain the time-frequency representation,

(1) $$y\left(n,k\right)=\overset{L-1}{\underset{m=0}{\sum }}y\left(n+m\right)h\left(m\right)e^{-j2\pi \frac{mk}{N}}$$

where s(·) represents the demodulated echo data, n=0,⋯,N−1 denotes the time index, k=0,⋯,K−1 is the discrete frequency index, h(·) is a Hanning window with length L. An example spectrogram of flipping fingers is illustrated in Figure 1. The length of Hanning window is set to 64 (0.064 s), the overlap of two consecutive windows is 63, and K is set to 256.

It can be clearly observed that most parts of the spectrogram are populated by background noises with relatively weak energy. Only in several localized and concentrated regions does the spectrogram possess stronger energy. The observation in fact states that the time-frequency distribution of dynamic hand gestures presents not merely completely sparseness features as discussed in ref. [27]. More precisely, it exhibits obvious local clustering characteristics, which can be more perfectly approximated by structured sparsity models.

It has been well studied and proven that sparsity based micro-Doppler features extraction methods can be of great benefit for HGR task in limited dataset scenarios [27,28]. However, no related work so far was reported to consider the clustering nature of the spectrograms of dynamic hand gestures.

### 2.2. Sparsity Model of Dynamic Hand Gesture

Firstly, we present the definition of a K-sparse signal. If a signal $x\in C^{M}$ can be approximated by K≪M non-zero coefficients under certain transformation, the signal is a called a K-sparse signal. Compressed sensing (CS) theory states that if the signal is sparse in a certain domain, the original signal can be accurately recovered using sparse reconstruction technique with a reduced observation [25].

Under complete sparsity hypothesis, by denoting the raw radar echoes of dynamic hand gestures as $y\in C^{N}$, the following sparse representation of y in time-frequency domain holds,

(2) y=Φx

where $\Phi \in C^{N*M}$ represents a time-frequency dictionary,$\;x\in C^{M}$ is a sparse vector. The above model states that the radar echo of dynamic hand gesture can be approximated using linear superposition of a series of basis signals, which can have various forms. This paper adopts the Gaussian-windowed Fourier basis signal [31], which can be expressed as,

(3) $$\Phi \left(n,m\right)≜\Phi \left(n|t_{m},f_{m}\right)=\frac{1}{2^{\frac{1}{4}}}exp\left[-\frac{{\left(n-t_{m}\right)}^{2}}{\sigma^{2}}\right]exp\left(jf_{m}n\right)$$

where $t_{m}$ and $f_{m}$ stand for the time and frequency shift of the basis signal, respectively, and σ denotes the variance of the Gaussian window, namely, the scaling factor. And n=1,⋯,N is the time shift index, while m=1,⋯,M denotes the frequency shift index.

The parameter σ is usually selected based on experience. Here, we set it to be 16. The values of $t_{m}$ and $f_{m}$ are empirically set to be {0.25σ, 0.5σ, 0.75σ,⋯,0.25σ×N/(0.25σ)} and $\left\{\frac{1\pi }{4\sigma },\frac{2\pi }{4\sigma },\frac{3\pi }{4\sigma },\cdots ,2\pi \right\}$, respectively, where ⌊·⌋ denotes a rounding down operation [31].

According to the theory of CS [32,33], for a K-sparse signal x, if *K* ≪ *N* < *M* holds, the sparse time-frequency distribution x in Equation (2) can be recovered by,

(4) $$\hat{x}=\underset{x}{\arg\min}{\left\|y-\Phi x\right\|}_{2}^{2},s.t.{\left\|x\right\|}_{0}\leq K,$$

where ${\left\|\cdot \right\|}_{0}$ and ${\left\|\cdot \right\|}_{2}$ denote the $L_{0}$ and $L_{2}$ norms, respectively. Equation (4) can be effectively solved using many algorithms [31,34,35]. Once the sparse coefficient is obtained, the raw radar echo of dynamic hand gestures can be approximated as follows,

(5) $$\hat{y}=\Phi \hat{x}=\overset{K}{\underset{k=1}{\sum }}{\hat{x}}_{ik}\Phi \left(n|t_{ik},f_{ik}\right)$$

**Figure 2:**
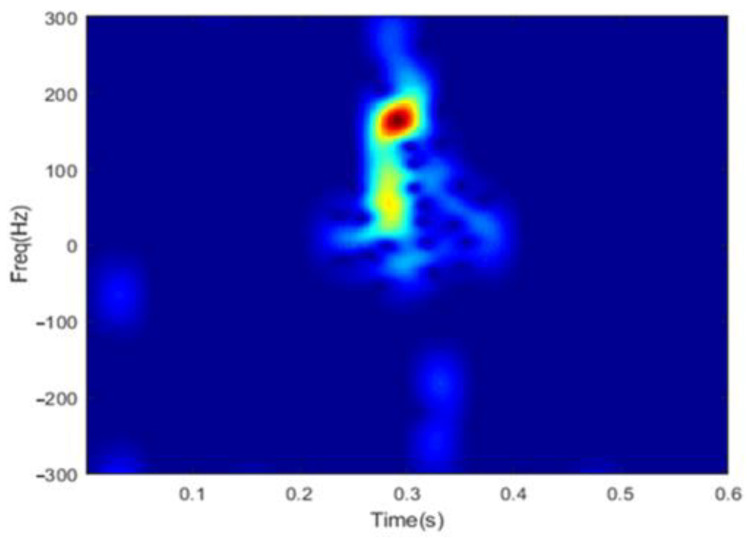
Spectrogram of reconstructed signal using the OMP algorithm.

The time-frequency distribution of the reconstructed radar echo $\hat{y}$ is shown in Figure 2, with K set to be 24. Compared with the raw spectrogram in Figure 1, it is obvious that the position and strength of the dominant time-frequency components are well preserved and highlighted. However, the noises are not removed perfectly. The reason is that the complete sparsity assumption is valid not only for time-frequency signatures of the hand gesture, but also for the noise components. Therefore, it is unavoidable that part of the noise components would be recovered as well in the reconstructed spectrogram.

### 2.3. Dynamic Group Sparsity Model of Dynamic Hand Gesture

In fact, the time-frequency distribution of dynamic hand gesture shows obvious clustering property while the noises tend to arbitrarily spread throughout the spectrogram. Meanwhile, the pattern of the cluster is not limited to any specific structure. Thus, we remodel the time-frequency distribution of dynamic hand gesture using a dynamic group sparsity model with more flexibility, in which an element surrounded by non-zero elements has a higher probability of being non-zero, and vice versa.

**Figure 3:**
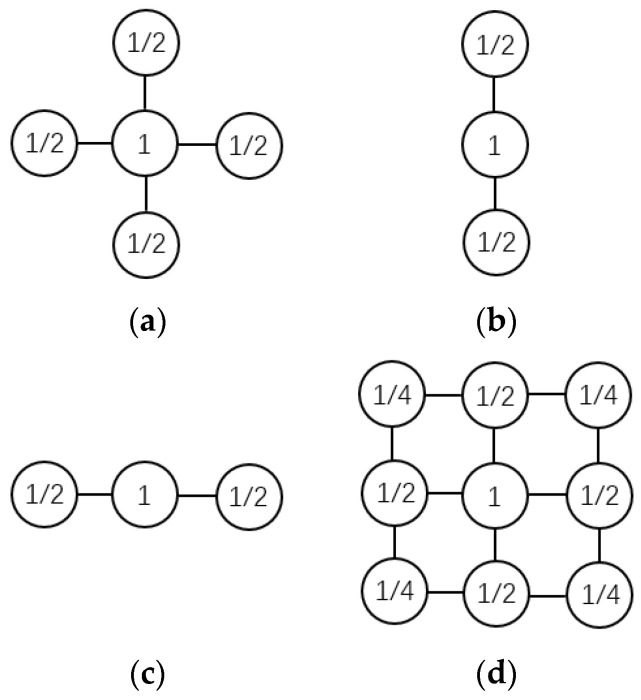
Group structures used in the DGS Pruning algorithm: (**a**) cross-shape structure, (**b**) vertical structure, (**c**) horizontal structure, (**d**) eight neighbors structure.

The dynamic group sparsity signal can be defined as follows: if a signal $x\in C^{M}$ can be approximated by K≪M non-zero coefficients under some linear transforms and these K nonzero coefficients are clustered into q∈{1,2,⋯,K} groups, the signal is called a dynamic $G_{K,q}$-sparse signal [30]. In this work, the group sparse representation of y in time-frequency domain is expressed as follows,

(6) $$y=\Phi x_{K,q},$$

where $x_{K,q}\in C^{M}$ is a group sparse vector. An effective algorithm, called Dynamic Group Sparsity-Subspace Pursuit (DGS-SP) [30], can be used to recover the above $G_{K,q}$-sparse signal, which is expressed as,

(7) $${\hat{x}}_{K,q}=\mathrm{DGS}-\mathrm{SP}\left(y,\Phi ,K,\beta \right),$$

where β denotes the weights of the neighbors. The most important feature of the DGS-SP algorithm is the introduction of a unique pruning process in the iteration, as described in Algorithm 1 below. Firstly, for the vector v needed to be pruned, the neighbor indices of each element are calculated. Then the neighbors are weighted and summed according to the weight coefficient β, and the result is recorded as z. The first K maximum values in z are taken as the pruned vector. After that, embedding the DGS pruning into the Subspace Pursuit (SP) algorithm results in the so-called DGS-SP algorithm. The pseudocodes of the DGS pruning and the DGS-SP algorithm are described in Algorithms 1 and 2, respectively. Different neighboring structures, namely the structured priors, can be adopted when conducting the pruning, as shown in Figure 3 [36,37], which comes with different recognition performance. It will be detailed in Section 4.

**Algorithm 1.** DGS pruning.
 **Input:** Signal $v\in R^{M}$, sparsity K, weights for neighbors β

 **Output:** solution support supp{v,K}

 **Step:**

  (1) compute the index $N_{x}\in R^{M*I}$ of the corresponding neighbors, I is equal to the number of non-zero elements in β;  (2) compute the weights $w=\left[\beta_{1},\beta_{2},\cdots ,\beta_{I}\right]$, $\beta_{i}={\left[\beta_{i},\beta_{i},\cdots ,\beta_{i}\right]}^{T}$
**for**
m=1
**to**
M
**do**  (3) compute $z\left(m\right)=v^{2}\left(m\right)+\sum_{i=1}^{I}w^{2}\left(m,i\right)v^{2}\left[N_{x}\left(m,i\right)\right]$, **end for**  (4) let supp{v,K} be the index corresponding to the first K maximum values in z.

**Algorithm 2.** DGS-SP.
 **Input:** Sparsity K, observation matrix Φ, original signal y, weight for neighbors β

 **Output:** Sparse approximation $\hat{x}$

 **Initialization:**

  (1) the residual $r^{0}=y$;  (2) the solution support $S^{0}=\emptyset$;  (3) the atom set $\varphi^{0}=\emptyset$;  (4) the iteration index l=0;
 **Iteration:** At the lth iteration, go through the following steps:

  (1) l=l+1;  (2) compute $v^{l}=r^{T}\Phi$;  (3) $\Omega =\mathrm{DGSPruning}\left(v^{l},K,\beta \right)$;  (4) $\;S^{l}=S^{l-1}\cup \Omega$;  (5) $\;\varphi^{l}=\Phi_{S^{l}}$;  (6) compute $b=arg\underset{{\hat{x}}^{l}}{\min}\|y-\varphi^{l}\hat{x}\|{}_{2}$;  (7)  Ω=DGSPruning(b,K,β);  (8) $\;S^{l}=S_{\Omega }^{l}$;  (9) $\;\varphi^{l}=\Phi_{S^{l}}$;  (10) compute ${\hat{x}}^{l}=arg\underset{{\hat{x}}^{l}}{\min}\|y-\varphi^{l}\hat{x}\|{}_{2}$;  (11) update $r^{l}=y-\varphi^{l}{\hat{x}}_{l}$;  (12) if $\|r^{l}\|{}_{2}\geq \|r^{l-1}\|{}_{2}$, quit the iteration.

**Figure 4:**
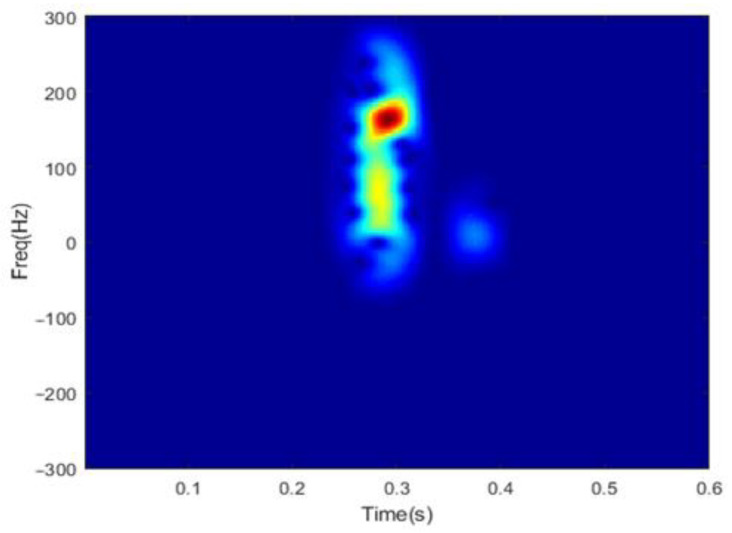
Spectrogram of reconstructed signal using the DGS-SP algorithm.

The time-frequency distribution of the reconstructed radar echo $\hat{y}$ using DGS-SP algorithm is shown in Figure 4. The group sparsity level is set to 24, which is consistent with the level in Section 2.2. The third structure as shown in Figure 3c is selected as the neighboring structure. As compared to the results in Figure 2, it is obvious that the micro-Doppler signatures of flipping fingers are well recovered while the noise components being significantly suppressed, which indicate that the proposed approach possesses better noise-isolation performance than the OMP algorithm.

### 2.4. Feature Extraction of Dynamic Hand Gesture

It was detailed in Section 2.2 that the time-frequency distribution of the hand gesture echo y can be approximated by a group of basis signals with parameter sets $\left(\left|x_{ik}\right|,t_{ik},f_{ik}\right)$, where $\left|x_{ik}\right|$ denotes the intensity of the specific time-frequency cell $\left(t_{ik},f_{ik}\right)$. Thus, the sets in effect serve to be representative features directly related to the data content of different hand gestures. By extracting such the discrete parameter sets of the pre-designated K-sparsity signal, the feature vectors can be formulated as below and utilized for subsequent hand gestures recognition.

(8) $$f\left(y\right)=\left(t_{i1},\cdots ,t_{iK},f_{i1},\cdots f_{iK},\cdots ,\left|x_{i1}\right|,\cdots \left|x_{iK}\right|\right)$$

**Figure 5:**
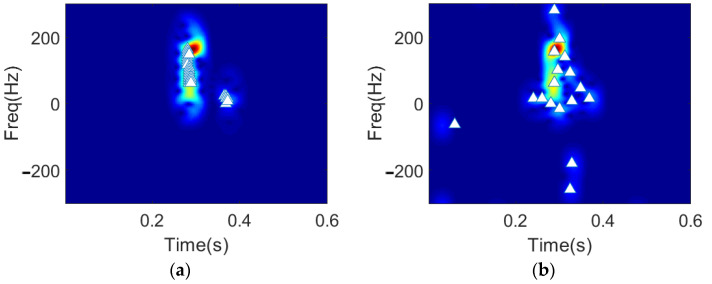
Demonstration of the feature vectors and the corresponding time-frequency distribution, yielded by: (**a**) DGS-SP, (**b**) OMP.

Then, we turn to the recovered spectrograms for a visually intuitive comparison of the distribution of the extracted features using the proposed method and the OMP-based method. In Figure 5, the white triangles in the spectrogram represents the time-frequency locations $\left(t_{ik},f_{ik}\right)$ of the extracted feature vector. As can be observed from the results, the features selected by DGS-SP algorithm are more focused around the major micro-Doppler signatures than that of the OMP method. Since the major micro-Doppler signatures contribute to the discrimination of different hand gestures it implies that the extracted feature vectors using the proposed approach can achieve better hand gesture recognition performance.

## 3. Results

### 3.1. Data Collection and Feature Extraction

The dataset of dynamic hand gestures utilized in this paper was collected using a software defined radar, SDR-KIT 2400T2R4, developed by Ancortek Inc., USA [38]. The platform is composed of two transmitters and four receivers. It can work either in frequency modulated continuous wave (FMCW) mode with the operating frequency ranging from 24 GHz to 26 GHz or in a single-tone CW mode with the frequency fixed at any intermediate value. We in this work used a laptop connected to the Ancortek millimeter wave radar to record and process the radar echoes of hand gestures. And the Python programming language is employed to implement all related signal processing method.

**Figure 6:**
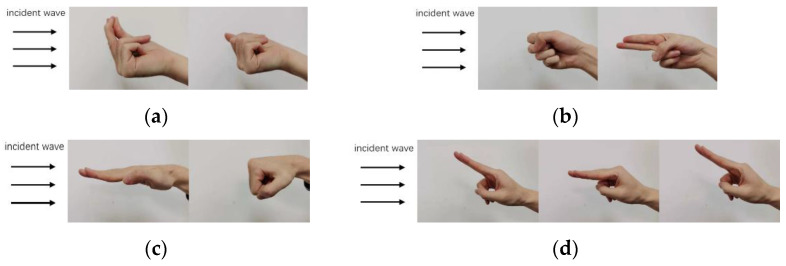
Illustrations of four different dynamic hand gestures: (**a**) snapping fingers, (**b**) flipping fingers, (**c**) clenching hand, (**d**) clicking fingers.

**Figure 7:**
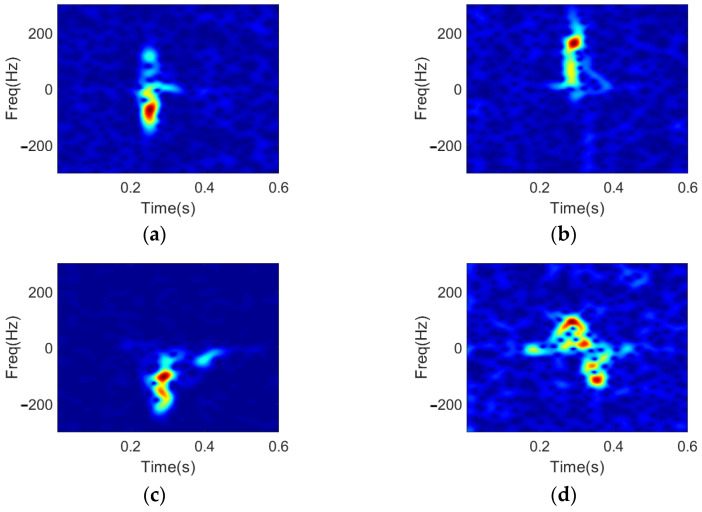
Spectrograms of received signals corresponding to four dynamic hand gestures: (**a**) snapping fingers, (**b**) flipping fingers, (**c**) clenching hand, (**d**) clicking fingers.

In the experiment, we use only one tranceiving antenna pair to collect the scattered data. The data acquisition setup and the schematic diagrams of four dynamic hand gestures are illustrated in Figure 6. The radar system operates at 24 GHz with a sampling frequency of 1 kHz. The separation between the antenna front and the human hand is about 0.3 m. In total, four types of hand gestures are considered, including (a) snapping fingers, (b) flipping fingers, (c) clenching hand, and (d) clicking fingers. Four human subjects were recruited to conduct the experiment. Each of them repeated the four gestures for 25 times. The duration of radar recording lasts for 15 s. Thus, a complete dynamic hand gesture cycle is about 0.6 s. In this way, we can obtain 400 records of four hand gestures, 100 for each. Then we use the same parameters as introduced in Section 2.1 to calculate the spectrograms of the four types of dynamic hand gestures, and the results are shown in Figure 7.

**Figure 8:**
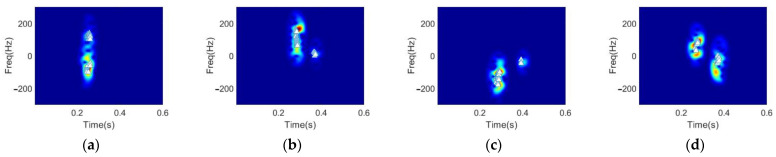

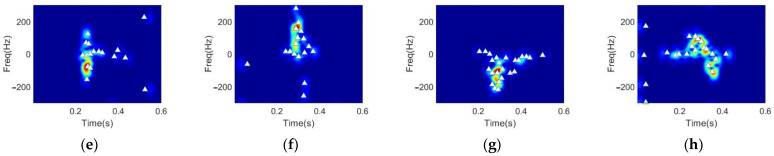
Demonstration of the feature vectors and the corresponding time-frequency distributions of the four hand gestures by DGS-SP: (**a**–**d**), and by OMP: (**e**–**h**), with *K* = 24.

The reconstructed spectrograms and the feature vectors are shown in Figure 8, by using the OMP method and the proposed method, respectively, with the same parameters described in Section 2. The results show that the DGS-SP method, by modeling clustering characteristics of spectrograms of hand gestures using structured prior, has better performance in extracting key hand motion information and suppressing noises in spectrogram.

### 3.2. Recognition Accuracy Using Different Classifiers

**Figure 9:**
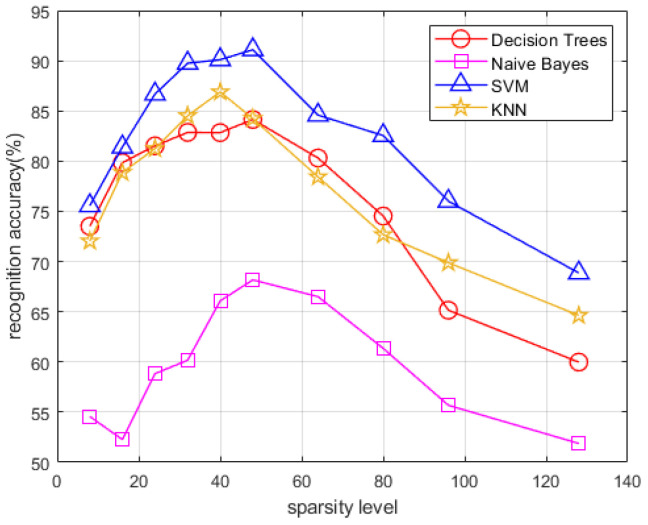
Recognition accuracy of the proposed method using different classifiers versus different sparsity levels.

Next, we consider the recognition accuracy. Each record of dynamic hand gesture was processed using the proposed method for each sparsity level. By repeating the above process for all the collected data samples, a dataset of feature vectors of different dynamic hand gestures of different sparsity levels is constructed. Then, several traditional machine learning classifiers are separately employed for recognition, including the Decision Trees, Naïve Bayes Classifiers, support vector machine (SVM) and K-nearest neighbor (KNN). Eighty percent of the dataset is selected as the training set, and the rest is used as the testing set. One hundred Monte Carlo trials are performed to produce an average recognition accuracy for each sparsity level. The mean recognition accuracy for four types of hand gestures obtained using different classifiers under various sparsity levels are depicted in Figure 9.

Clearly, SVM performs best for all the four types of hand gestures and thus is chosen as our classifier. Such a selection means that the maximum inter-class distance is obtained using the specific classifier, and thus it is more competent for the hand gesture recognition task when compared to other classifiers. The highest recognition accuracy rate reached 91.3% with sparsity level being set to 48.

### 3.3. Recognition Accuracy under Different Dynamic Group Structures

**Figure 10:**
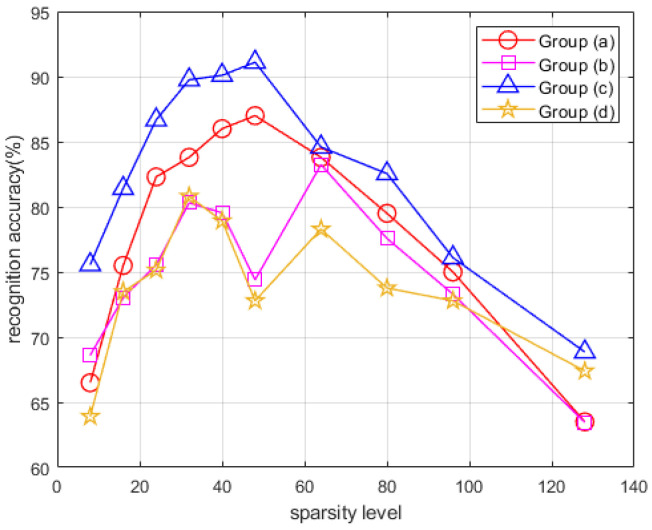
Recognition accuracy of the proposed method under different neighboring structures versus different sparsity levels.

The neighboring structure plays a key role in the DGS-SP method, which is also the key to distinguish it from the OMP method. Different neighboring structures can result in different reconstruction results, which will ultimately affect the classification performance. The results corresponding to different group structures (see Figure 3) are illustrated in Figure 10.

Among the four different neighboring structures, the type (c) structure yields an overall highest recognition accuracy. The underlying rationality lies in the fact that the time-frequency distributions of hand gestures exhibit a more evident vertical expansion rather than lateral expansion due to the high sensing frequency and the short time duration when conducting hand gestures. Therefore, it is more desirable to apply such a neighboring structure with prominent lateral expansion to preserve the information related to the time dimension. Thus, the group structure (c) is suggested to be exploited to model the clustering nature of the spectrograms of the dynamic hand gestures.

### 3.4. Comparison with the OMP Method

**Figure 11:**
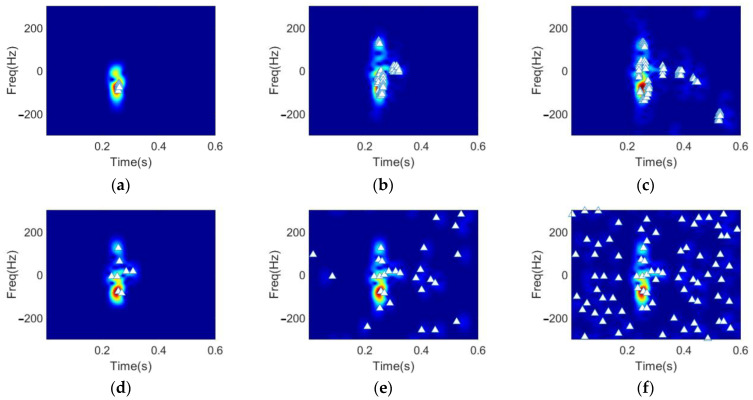
Feature vectors and the corresponding time-frequency distributions based on DGS-SP: (**a**–**c**), and OMP: (**d**–**f**), with the sparsity levels chosen as 8, 48, and 128, respectively.

The recognition performance of the proposed approach is given in this subsection, in comparison with the OMP-based method [27]. To give a more intuitively illustration, the reconstructed spectrograms of the snapping fingers under different sparsity levels using the DGS-SP approach and the OMP approach are first shown in Figure 11 with the selected feature vectors’ coordinates highlighted using white triangles in the spectrograms.

**Figure 12:**
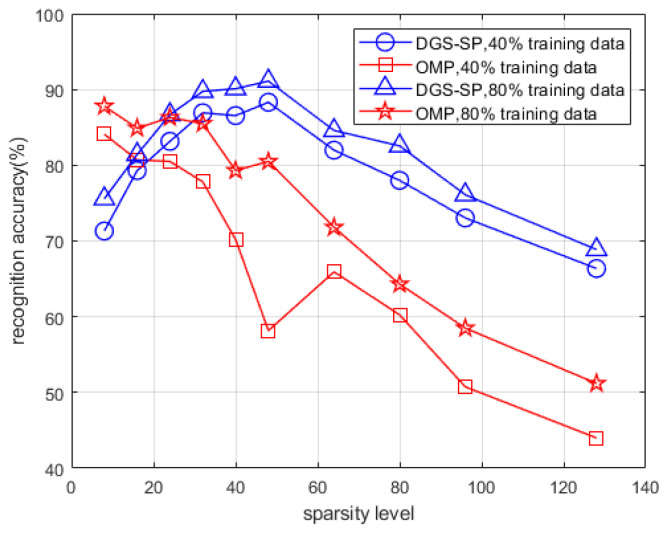
Recognition accuracy of the proposed method and the OMP method versus different sparsity levels.

Then, quantitative comparison via evaluating the recognition accuracy by using 40% and 80% of the data as the training datasets are given in Figure 12. It can be found that the proposed method outperforms the OMP-based method when choosing the sparsity level larger than about 25, meaning that the proposed method is more robust with respect to the sparsity levels. The proposed method yields the highest recognition accuracy of 91.1% (mean value for the four gestures) when the sparsity level is set to be 48, while that value for OMP is 87.8% with the sparsity level set to be 8.

Clearly, the proposed approach has the advantages of better anti-noising ability and flexibility in adaptations of structures of spectrograms. It thus outperforms the traditional complete sparsity approach. The corresponding confusion matrix in this scenario for the DGS-SP approach is depicted in Table 1.

### 3.5. Comparison with the CNN Method

**Figure 13:**
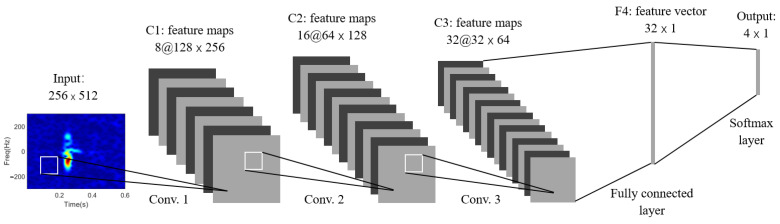
CNN architecture of three convolutional layers.

**Figure 14:**
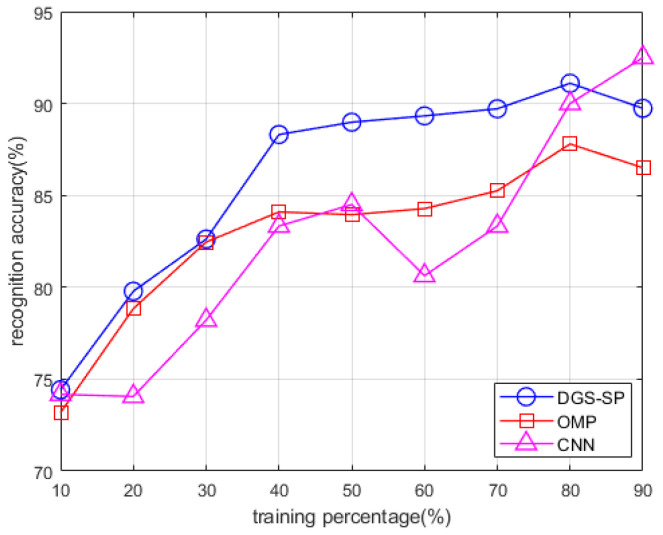
Recognition accuracy of the proposed method, the OMP method and the CNN-based method under different sizes of training set.

Finally, the recognition performance of the proposed method, OMP-based method and the convolutional neural network (CNN) method, another widely adopted approach [39] as illustrated in Figure 13, is analyzed with different sizes of training dataset. The size of the training data varies from 10% to 90% with a step of 10%. The sparsity level is set to be 48 for the proposed method, and the value for OMP is chosen as 8 (the best parameter for each one, as shown in Figure 12). The results are shown in Figure 14.

Note that the recognition performance of the proposed method is better than that of OMP. Meanwhile, in the case of small dataset, the proposed method achieves higher recognition accuracy than the CNN method.

## 4. Conclusions

In this paper, we investigated and exploited four structured prior of the time-frequency distributions of dynamic hand gestures in order to enhance the hand motion features and further improve the recognition performance. A dynamic group sparsity model and DGS-Subspace Pursuit algorithm was utilized to model the spectrograms of the hand gestures. Such a modeling can well isolate the features of dynamic hand gestures and the noise components in the spectrograms. By experiment, we choose SVM as the classifier and sparsity level is set to be 48. Experimental result shows that the proposed method improves recognition accuracy about 3.3% over the OMP-based method and has a better performance than CNN based method in small dataset. The experiment demonstrated the effectiveness of the proposed method. The method proposed in this work is not only suitable for the recognition of simple hand gestures, but also for the recognition of more complex sign language gestures, which can enhance the feature extraction process. For future work, we would like to exploit dual-hands’ gesture recognition and more subtle and complex sign language gesture using phased array radar or smart metasurface. And for the limitation of hardwares, we feel that the commercial millimeter wave radar still has many places to improve, such as the operating distance improvement under the low power illumination, the antenna array configuration that enables user and environment sensitive and configurable beam radiation, etc.

## Figures and Tables

**Table 1:** Confusion matrix of the proposed method.

	Snapping Fingers	Flipping Fingers	Clenching Hand	Clicking Fingers
Snapping fingers	81%	4%	7%	8%
Flipping fingers	1%	98%	0%	1%
Clenching hand	2%	0%	97%	1%
Clicking fingers	7%	4%	0%	89%

## Data Availability

Data sharing is not applicable to this article.
